# Growth of Focal Nodular Hyperplasia is Not a Reason for Surgical Intervention, but Patients Should be Referred to a Tertiary Referral Centre

**DOI:** 10.1007/s00268-017-4335-6

**Published:** 2017-11-22

**Authors:** Mirelle E. E. Bröker, Anne J. Klompenhouwer, Marcia P. Gaspersz, Annick M. E. Alleleyn, Roy S. Dwarkasing, Indra C. Pieters, Robert A. de Man, Jan N. M. IJzermans

**Affiliations:** 1 Department of Surgery, Erasmus Medical Centre, ‘s Gravendijkwal 230, 3000 CA Rotterdam, The Netherlands; 2 Department of Radiology, Erasmus Medical Centre, Rotterdam, The Netherlands; 3 Department of Radiology, VU University Medical Centre Amsterdam, Amsterdam, The Netherlands; 4 Department of Gastroenterology and Hepatology, Erasmus Medical Centre, Rotterdam, The Netherlands

## Abstract

**Background:**

When a liver lesion diagnosed as focal nodular hyperplasia (FNH) increases in size, it may cause doubt about the initial diagnosis. In many cases, additional investigations will follow to exclude hepatocellular adenoma or malignancy. This retrospective cohort study addresses the implications of growth of FNH for clinical management.

**Methods:**

We included patients diagnosed with FNH based on ≥2 imaging modalities between 2002 and 2015. Characteristics of patients with growing FNH with sequential imaging in a 6-month interval were compared to non-growing FNH.

**Results:**

Growth was reported in 19/162 (12%) patients, ranging from 21 to 200%. Resection was performed in 4/19 growing FNHs; histological examination confirmed FNH in all patients. In all 15 conservatively treated patients, additional imaging confirmed FNH diagnosis. No adverse outcomes were reported. No differences were found in characteristics and presentation of patients with growing or non-growing FNH.

**Conclusion:**

This study confirms that FNH may grow significantly without causing symptoms. A significant increase in size should not have any implications on clinical management if confident diagnosis by imaging has been established by a tertiary benign liver multidisciplinary team. Liver biopsy is only indicated in case of doubt after state-of-the-art imaging. Resection is deemed unnecessary if the diagnosis is confirmed by multiple imaging modalities in a tertiary referral centre.

## Introduction

Focal nodular hyperplasia (FNH) is a benign liver tumour with an incidence in the general population of 0.6–3% [1]. FNH is especially common in young women, with a male/female ratio of 1:12 [2]. So far, no explanation has been found for the gender bias; female hormones or the use of oral contraceptives do not seem to play a role in prevalence [3, 4].

An FNH lesion consists of benign hepatocytes surrounding a central fibrous scar with a prominent dystrophic artery. The underlying mechanism of FNH formation is thought to be due to a vascular malformation and injury [5]. Patients do not have an underlying liver disease and are mostly asymptomatic [6].

With the current availability of highly sensitive imaging techniques, FNH is diagnosed more often as an incidental lesion. Magnetic resonance imaging (MRI) with liver-specific contrast agents has a very high specificity of almost 100% in larger lesions (>3 cm) but is less accurate with a sensitivity of 70–80% to diagnose smaller lesions where the central scar may be missing. In these cases, the combination of MRI and contrast-enhanced ultrasound (CEUS) provides the highest diagnostic accuracy [7].

This year, the European Association for the Study of the Liver (EASL) issued the first clinical practice guideline for benign liver tumours [8] in which they state that treatment of FNH is not recommended because of the benign character of FNH, the low incidence of intralesional bleeding [9, 10] and the absence of malignant transformation [11]. In case of doubt about the diagnosis FNH, a biopsy may be considered [8]. The guideline describes treatment is only pursued in exceptional cases such as expanding FNH.

It has been documented that FNH lesions may show a slow and incidental increase in size during follow-up. However, change in size may cause doubt about the diagnosis and the benign character of the liver lesion [12]. Growth of FNH has been suggested to be an indication for resection [13–15], although evidence for this approach is weak. The aim of this study was to evaluate how often a FNH grows and what are the implications for management and compare the characteristics of those with and without growing FNH.

## Materials and methods

To evaluate the course of disease of FNH lesions increasing in size during follow-up, we performed a retrospective cohort study including all patients who had been diagnosed with FNH in the Erasmus University Medical Centre, a tertiary referral centre for focal liver lesions. Inclusion started in 2002, from the moment that we had the availability of two imaging techniques with high sensitivity and specificity to establish the diagnosis FNH, and ran until 2015. Diagnosis FNH had to be confirmed on at least two radiologic modalities, including at least one contrast-enhanced MRI and one contrast-enhanced CT scan or CEUS, and established in a multidisciplinary tumour board committee. Sequential imaging had to be available with at least a 6-month interval.

Baseline characteristics, including gender, age and body mass index (BMI), were collected from electronic patient records. Patients were scored as symptomatic if abdominal pain or general discomfort was reported in history. Information on the number and size of the FNH lesions was collected from radiological and histological reports. Data on clinical management were obtained from the reports of the multidisciplinary tumour board committee and correlated with data obtained from surgical, radiological and pathological reports.

The radiological reports of all patients were re-examined, and growth was established if an increase in size between the diagnostic scan (T1) and follow-up scan at least 6 months after the initial scan (T2) was found. The diagnostic and follow-up scans were reassessed independently by two experienced radiologists (R.D. and I.P) from two tertiary referral centres. Because of the imprecise measurements of size in small lesions and potential bias in outcome, patients with lesions < 20 mm in both diagnostic and follow-up scans were excluded. We defined growth as an increase in size of at least 20% according to the RECIST criteria for solid liver tumours [16], as no other criteria have been validated. To evaluate whether lesion growth was related to weight gain, additional thickness of the subcutaneous fatty layer in the abdominal wall was measured on initial and follow-up imaging. Measurements were performed by both radiologists separately in the midline (linea alba) on the level of the origin of the coeliac artery.

### Radiology

In patients with a diagnosis of FNH who were found to have an increase in size, the diagnostic and follow-up scans were reviewed. MR imaging was performed with 1.5-T MR systems using a standard MRI protocol of T1-weighted, T2-weighted sequences and a dynamic contrast-enhanced series after intravenous administration of a bolus of 30 ml of non-liver-specific gadolinium chelate (gadopentetate dimeglumine, Magnevist; Schering, Berlin, Germany). CT scans were performed with 16- and 64-detector machines with a multiphase CT protocol consisting of plain, arterial- and portal-venous-dominant phase scans of the liver after i.v administration of 120 cc (Visipaque, General Electric Healthcare Medical Systems, Milwaukee, Wisconsin, USA). The lesions were scored as typical FNH if they were lobulated, a central scar was present, the aspect of the lesion was homogenous on the diagnostic MRI conform generally accepted classical imaging features of FNH. If there was no consensus on diagnosis or MR imaging showed no typical FNH, pathological examination had to have been performed for patients to be included in this study. Size measurements were done after complete evaluation of the MRI with confident diagnosis of FNH assessed by the readers. After evaluation of all sequences, measurements of lesion size were performed on images that deemed most accurate for this purpose in the perception of the reader; both MRIs were measured in the same phase. In most cases, this was done on the T1W after i.v contrast infusion during the arterial-dominant phase. If imaging had been performed in collaborating hospitals according to our protocol, the outcome would have been reviewed in our hospital.

### Data analysis

All analyses were performed using the Statistical Package for the Social Sciences (SPSS) version 20.0 (SPSS, Chicago, IL, USA). Continuous variables were summarized as median and interquartile range and categorical data as *n* (%) in case of a denominator >50 or a proportion/*n* in case of denominator <50. Differences between groups were assessed using the Mann–Whitney *U* test for continuous variables and Chi-squared test for binary variables. Correlation between variables was analysed using Pearson product-moment correlation coefficient. Statistical significance was considered at a *p* value < 0.05.

## Results and discussion

**Fig. 1:**
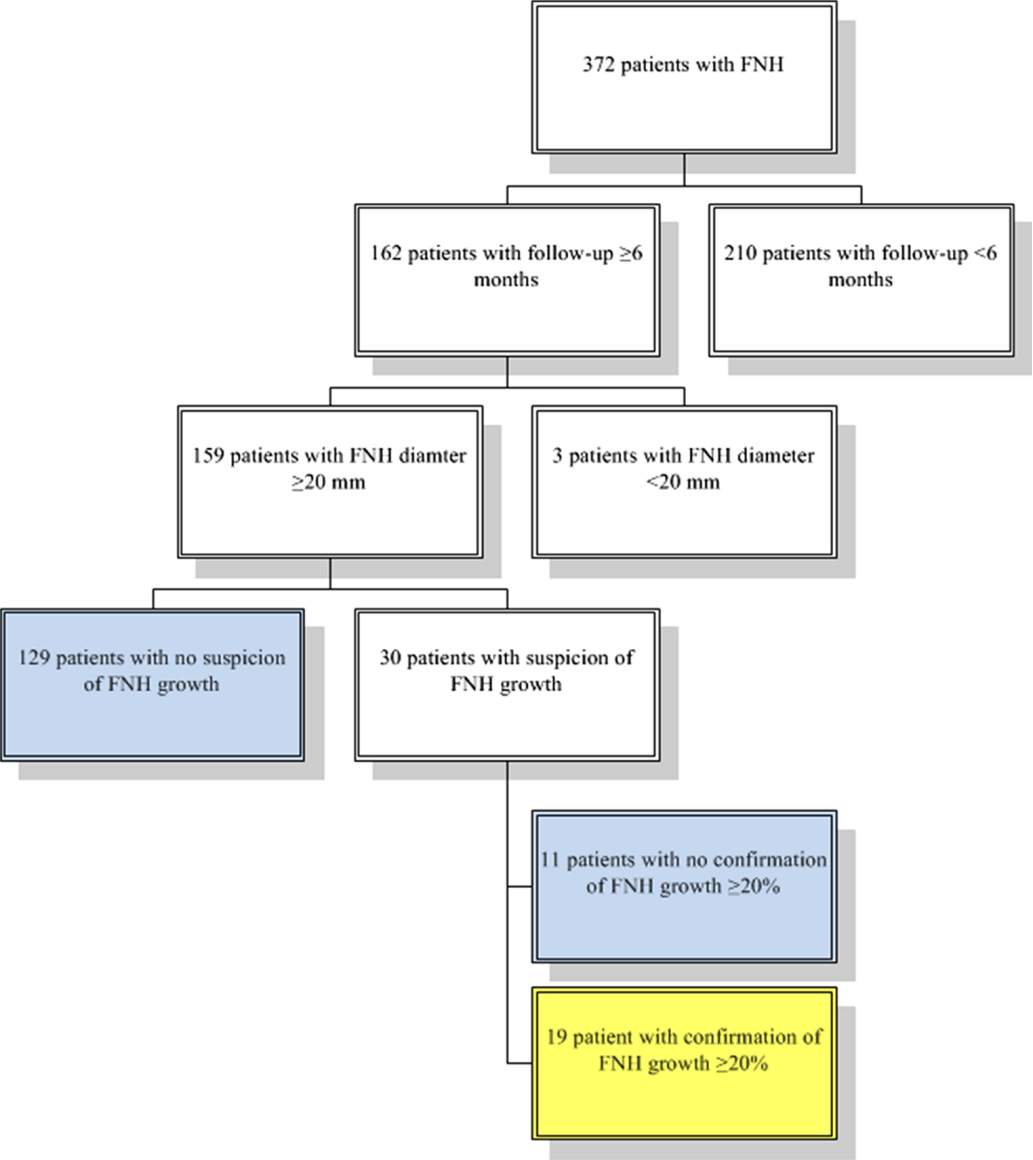
Flow chart inclusion

**Fig. 2:**
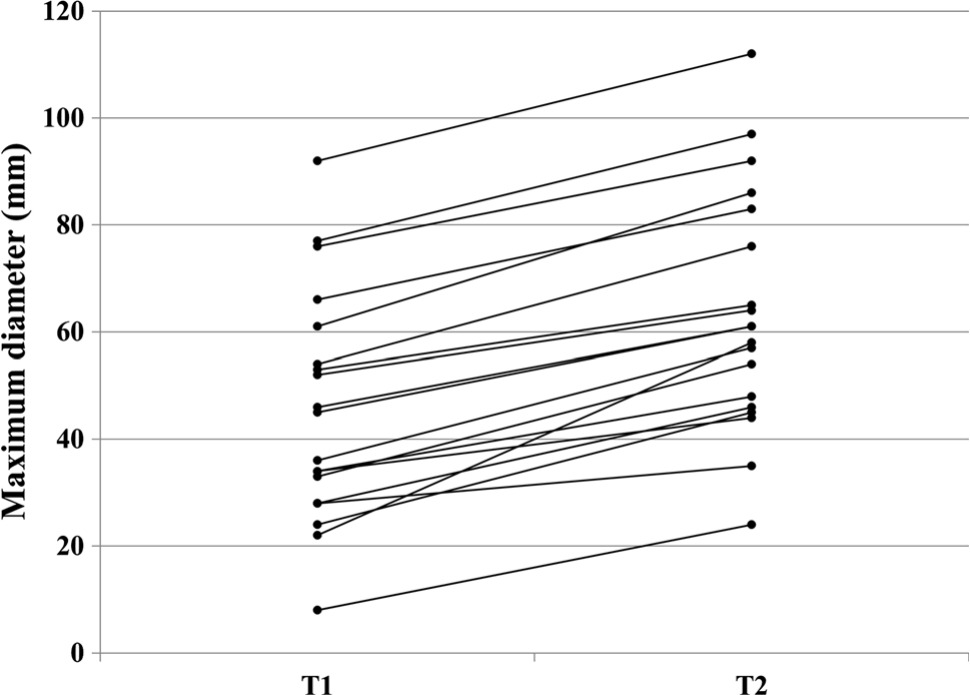
Example of a growing FNH. **a** FNH at T1: diameter 29 mm. **b** FNH at T2: diameter 55 mm

Out of 372 patients with a suspected FNH, 162 (44%) were included for growth analysis as sequential imaging was available with at least a 6-month interval (Fig. 1) and the remaining 210 patients were excluded. Because follow-up was less than 6 months, they were discharged when diagnosis FNH was established. Three patients were excluded from growth analysis because the maximum diameter of the lesion was <20 mm on both diagnostic and the follow-up scan. The diagnosis FNH was confirmed by the two radiologists in all cases. In 160 patients, the diameter measurements from the first (T1) and last (T2) radiological reports were examined, and in 28 patients (18%), an increase in size was found. Confirmation of increase with at least 20% was obtained in 19/28 patients as defined by both radiologists (Fig. 2).

Patients with growing and non-growing FNH did not differ regarding gender, age, BMI, number of lesions, symptoms or use of oral contraceptives (Table 1). The number of patients who underwent surgery or embolization of FNH, and underwent follow-up for at least 6 months, was significantly higher in the growing FNH group compared to the non-growing FNH group (11 and 5%, respectively, *p* = .009) although these patients had no complaints. No adverse events occurred in the patients with an FNH, including patients with growing FNH who did not undergo treatment.

**Table 1:** Patient and lesion characteristics

	Growing FNH (*n* = 19)	Non-growing FNH (*n* = 143)	*p* value
Female	19/19	137 (96%)	.363
Age	33 (24–42)	34 (27–43)	.248
BMI	25.5 (24–29)	24.7 (22–30)	.351
Lesions			.677
Solitary	12/19	76 (53%)	
Multiple	7/19	67 (47%)	
Symptoms			.962
None	5/19	38 (27%)	
Upper abdominal pain	10/19	73 (51%)	
Atypical complaints	3/19	18 (13%)	
Elevated liver enzymes	1/19	10 (7%)	
Unknown	0/19	3 (2%)	
Treatment			.009
No	15/19	136 (95%)	
Yes	4/19	7 (5%)	

Diagnostic biopsy was performed in 18/162 patients (11.1%): four histological examinations were inconclusive, and 14 confirmed the diagnosis FNH. Indications for biopsy were growth in four and uncertainty about the diagnosis on imaging in 14.

In total, 11/162 (6.7%) patients underwent resection (*n* = 9) or embolization (*n* = 2) of FNH. In all resected cases, the diagnosis FNH was confirmed by histological examination of the specimen. In 4/9, the radiological diagnosis was uncertain, and in the remaining 5/9 patients, the reason for resection was abdominal pain or dyspepsia. Abdominal pain only resolved in one patient who underwent treatment because of symptoms thought to be caused by FNH, in the remaining four patients the surgery did not provide symptom relief. The indication for embolization was abdominal pain in both patients; neither of them experienced symptom relief.

### Growing FNHs

**Fig. 3:**
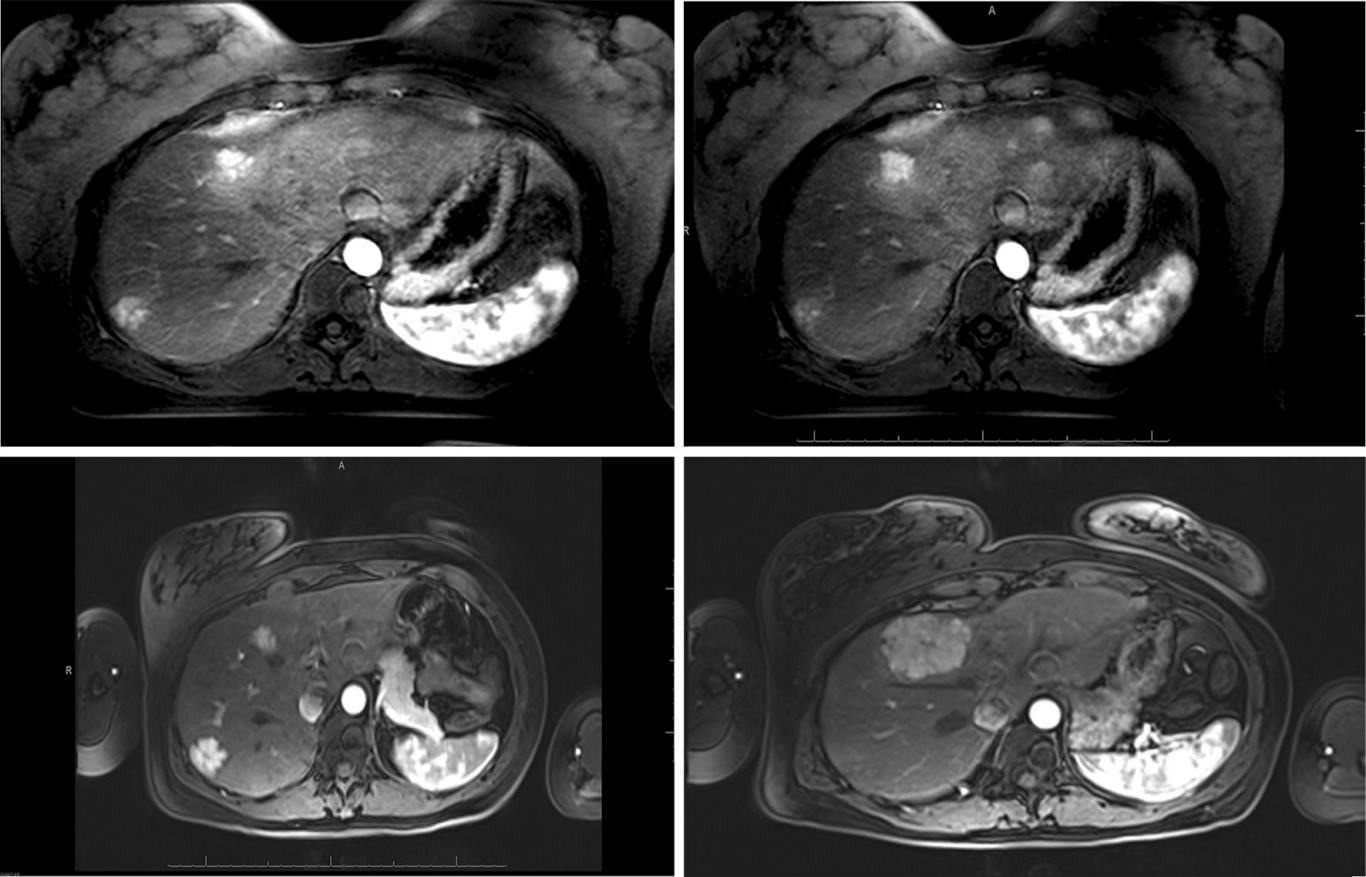
Size-growing FNH. This figure shows T1 (**a**; diagnostic scan 2009) and T2 (**b**; follow-up scan 2012) of the FNH in which growth is confirmed

Characteristics of growing FNH are summarized in Tables 2 and 3. In the growing FNH group, the median follow-up time was 31 months (IQR 25–42). Growth percentage ranged from 21.1 to 200% (Fig. 3). The majority of lesions (10/19) were located in the right hemiliver, and 9/12 were left-sided. Four patients underwent resection: three is because growth caused doubt about the diagnosis and one because of a symptomatic lesion. Three resected FNHs were located in the right lateral liver, and one in the left lateral liver. Pathology reports of the resected lesions all confirmed benign FNH. None of the patients who underwent resection had a diagnostic biopsy of the lesion before surgery.

**Table 2:** Lesion characteristics of growing FNH

Patient	Time between imaging sessions (weeks)	Number of lesions	Maximum diameter first imaging session (mm)	Maximum diameter last imaging session (mm)	Percentage increase T1–T2 (%)	Increase subcutis mm (%)
1	136,148	7	34*26	44*37	29.4	20.50 (141%)
2	137	3	76*58	92*64	21.1	2.50 (14%)
3	149	1	35*25	57*47	62.9	− 1.00 (− 13%)
4	319	1	61*57	86*74	41.0	− 4.50 (− 13%)
5	185	1	8*7	24*23	200.0	.50 (2%)
6	118	1	77*71	97*87	26.0	− .50 (− 2%)
7	258	5	66*48	83*53	25.8	3.00 (9%)
8	235	1	54*46	76*65	40.7	− 1.00 (− 3%)
9	135	1	28*24	35*31	25.0	5.50 (46%)
10	151	2	22*21	58*43	163.6	− 4.50 (− 21%)
11	111	1	45*36	61*52	35.6	4.00 (24%)
12	50	1	53*36	65*49	22.6	13.50 (75%)
13	137	1	34*30	48*45	41.2	3.00 (15%)
14	115	1	33*24	54*40	63.6	7.50 (26%)
15	108	1	46*34	61*50	32.6	6.50 (25%)
16	53	1	28*33	46*40	64.3	− 5 (− 9%)
17	164	2	92*60	112*68	21.7	− 1 (− 5%)
18	435	2	24*21	45*44	87.5	− 1 (− 7%)
19	118	1	52*41	64*46	23.1	4 (11%)

**Table 3:** Summary of characteristics of growing FNH

Median follow-up time (months)	31 (IQR 25–42)
Location	
Right hemiliver	10
Left hemiliver	9
Conservative treatment	18/19
Resection	4/19
Doubt about diagnosis due to growth	3
Symptomatic lesion	1

In all 15 patients treated with a wait-and-see policy, additional imaging was performed (MRI with liver-specific contrast or CEUS) which confirmed the lesions to be FNH. Thirteen out of these 15 were discharged from follow-up or were referred back to their initial hospital; two patients were kept in follow-up every 2–3 years according to their own wishes.

There was no statistically significant correlation between the growth percentage of the FNH and the percentage difference in subcutaneous fat (*r* = − .214, *p* = .340).

## Discussion

This study reports on a large series of patients with FNH and their follow-up. A specific focus of attention in our study was to evaluate if growth of FNH should have implications on clinical management, as growth may cause doubt about the initial diagnosis. In our study population, 12% of the lesions showed growth over a period of at least 6 months. It should be noted that this figure most probably overestimates the incidence of growing FNH and there may be a bias in observation as the patients included in our analysis were referred to a tertiary referral centre because of uncertainty about the diagnosis and management.

The diagnosis FNH was confirmed by resection in four patients and additional imaging in the form of MRI with liver-specific contrast agents or CEUS in the rest of the patients. No adverse events were reported in the group of growing FNHs. In line with the studies of Weimann et al. [15] who observed five patients with growing FNH and Perrakis et al. [14] who described 13 patients with growing FNH, we were unable to identify risk factors for growth.

In the 18 biopsies that were performed in our cohort, 14 (77.8%) confirmed FNH, while in a recent study from Sannier et al. a diagnostic accuracy of 95% in 19 patients was reported [17]. This could be explained by the fact that the accuracy for histologically diagnosing FNH and especially the distinction from other solid liver tumours such as hepatocellular adenoma and hepatocellular carcinoma has improved significantly in the study period. In 2009, Bioulac-Sage et al. [18] published a paper in which they were the first to describe abundant expression of glutamine synthetase as a marker to distinguish FNH from other hepatic lesions.

Our results suggest that growth of FNH is quite common and that growth in itself should not have any implications for clinical management. Growth may cause doubt about the initial diagnosis; but if imaging characteristics are typical for FNH, this is not necessary. MRI with liver-specific contrast agents in combination with CEUS has the highest accuracy for FNH diagnosis [19–21]. Growth on itself may not be an indication for biopsy: in our centre the final recommendation on whether or not biopsy is deemed necessary is made in a multidisciplinary liver tumour board meeting. In general, our recommendation is to only perform a biopsy when a discrepancy in diagnosis exists between the two imaging modalities.

It must be noted that the accuracy for diagnosing FNH with MRI has improved significantly in the study period. As of 2008, gadolinium-based contrast agents were used, making distinction from hepatocellular adenoma more accurate [22]. This could imply that some of the tumours were inadequately diagnosed as FNH before 2008. However, by including only tumours that were diagnosed based on two imaging modalities (MRI and CEUS), this proportion was kept to a minimum. In the future, additional analysis on the performed MRI with liver-specific contrast could be performed. The method of choice of liver-specific contrast is gadobenate dimeglumine. Another liver-specific contrast agent that might be used is gadoxetic acid (Primovist, Bayer Schering Pharma, Berlin, Germany). Quantitative analysis of the uptake of this liver-specific contrast could help for the differentiation between HCA and FNH. Grieser et al. showed the relative enhancement and liver-to-liver enhancement of HCA were lower in HCA compared to FNH with the use gadoxetic acid (Grieser 2013/2014) and might be the most recent method of choice for FNH. Due to the inclusion period and the use of Gadolinium chelate, additional analysis is not performed and is one of the limitations of this study [23, 24].

Differences in management between FNH and hepatocellular adenomas demand an accurate differentiation. Resection is indicated for hepatocellular adenoma if the tumour exceeds a diameter of 5 cm 6 months after the use of Oral Contraceptive is stopped, because of the risk of bleeding [25]. In contrast, for FNH, no strict indications for resection are defined. As liver resections may have a peri-operative complication rate up to 20–25%, a diagnostic liver resection is not advisable [26]. In the case of FNH, the liver resections are generally performed in young, healthy women. As our study showed no complications of the conservative approach, we advise to avoid resection as described in the EASL clinical practice guideline [8], even if the lesion is growing.

FNH is often an incidental finding discovered by various imaging techniques. In our cohort, we found that 26.5% of the patients were asymptomatic, while most studies have shown a large percentage of asymptomatic patients ranging from 65 [14] to 90% [27]. One possible explanation could be that the Erasmus Medical Hospital is a tertiary referral centre, and more patients with symptoms are referred. We assume that most of the symptoms are not caused by the presence of FNH and that FNH indeed could be asymptomatic. If treated, patients need to be comprehensively informed and it should be stressed that it may not be guaranteed that the abdominal pain will resolve [28].

The biggest limitation of our retrospective study is the design that is inherent to bias. In addition, it may be questioned whether the sample size of the growing FNH group is large enough to justify the conclusion; however, with 19 patients, we are the first to describe such a series of growing FNH and others may be challenged by this report to add new data.

In conclusion, our series confirm that FNH is not a static lesion and that growth may occur rather frequently. It must be noted that patients with a growing FNH do not report more pain or discomfort compared to the patients with non-growing FNH. Moreover, growth in itself should not have any implications on clinical management. In case of doubt, MRI with liver-specific contrast agents in combination with CEUS provides the highest diagnostic accuracy. As these imaging techniques are not available in every hospital, patients could be referred to a centre specialized in focal liver lesions. Growth is not an indication for liver biopsy, and biopsy should only be considered when the two imaging modalities do not provide the same diagnosis.

This study shows that FNH may grow significantly without causing symptoms. No adverse outcomes were observed in patients with growing FNHs. Therefore, we recommend, and use this in our hospital nowadays, that even-growing FNHs should not be resected and follow-up (growing) of FNH after a certain diagnosis made in a tertiary referral centre is not indicated.
